# A Course of Clinical Lectures, Delivered at the Hôtel Dieu, Paris, for the Session 1842-’43

**Published:** 1843-09-16

**Authors:** A. F. Chomel

**Affiliations:** Paris


### THE MEDICAL EXAMINER,

### And Retrospect of the Medical Sciences.

Vol. VI.] PHILADELPHIA, SATURDAY, SEPTEMBER 16, 1843. [No. 18.

### A COURSE OF CLINICAL LECTURES

*Delivered at the Hôtel Dieu, Paris, for the Session 1842–'43.*

BY A. F. CHOMEL, M. D.

### LECTURE XII.

RESUMÉ OF THE ANNUAL CLINIC.

(Continued.)

*Diseases of the Uterus and of the Hypogastric Region.*

We have had thirty-four cases of phlegmasiæ of the organs of this region. Under this head is not only included those affections of the uterus and its appendages following delivery, but also those due to causes unconnected with the puerperal state. Except six cases, all the others followed directly or indirectly this state. I have had frequent occasion to remark, and repeat it again at this time, on the great dangers which women incur in quitting too soon those establishments in which they have been confined. Of this fact we have had incontestable proof in the numerous cases which have fallen under our observation. Thus, of eight women who died of puerperal metritis, seven left the hospital before the seventh day, and one on the eighth. The danger of two early exposure to newly delivered women is so great, that it ought to awaken the attention of the administration of hospitals, and regulations should be enacted which would prevent these unfortunate women from leaving the hospitals before they were in a condition to attend to their affairs. You must snatch them, in spite of themselves, from certain danger.

The symptoms which announce this affection are, at the commencement, a chill, then ill-defined pains in the hypogastric region, the foetid smell of the lochia, more or less fever, general prostration of the forces, &c. &c. When these symptoms are present, you have every reason to suspect the commencement of uterine disease, or of some phlegmasia of the appendages of the organ. When the uterus is sensitive to the touch, and more voluminous than it should be naturally, there can be no doubt of its condition. Sometimes the broad ligaments and the surrounding cellular tissue is the seat of inflammation. In this case, if you explore the parts by an examination per vaginam, through the abdomen, and by the introduction of the finger into the rectum, you will distinguish a tumour more or less elastic, or fluctuating, and which sometimes terminates in resolution, but more frequently opens either into the rectum, bladder, or sometimes vagina. Whenever the physician observes the symptoms which I have just mentioned, joined to active febrile disturbance, which cannot be otherwise accounted for, he should immediately direct his attention to these parts. The diseases in question are sometimes developed in a latent form; at other times they simulate, at the commencement, rheumatic or neuralgic affections, by pain in the neighbourhood of the large joints, and along the course of the nerves, or large vessels. Sometimes at the same time with pain, swelling of the larger extremities occur. The cause of these symptoms is not too well known; but, very commonly you will find on a careful examination, a tumour in the pelvis, or elsewhere, pressing on the great venous trunks. You meet with the same symptoms frequently in men, who suffer from tumours of the rectum. You also see retractions of the extremities, sciatic neuralgias, which can be referred to no other cause. Thus then, these diseases are of highly practical interest, for it is only by an exact acquaintance with the precise nature of these affections that you can employ suitable remedies. Among the facts of this kind there are several very remarkable ones. Among others I recall the case of the woman who died, and in whom we found a tumour in the hypogastric region, communicating with a sac, and with the uterus itself, and gangrened to a considerable extent. This patient, at her entrance, was in a state of considerable febrile excitement, with a fetid discharge from the external organs of generation. After several days of treatment she improved, but of a sudden all her symptoms became aggravated, pus was discharged by the intestines, and the condition of the patient became worse and worse, until she died. At the autopsy we found the local lesions that I have just mentioned. It was difficult to explain, in a satisfactory manner, the connection of these disorders with the symptoms observed during life, and to know if the gangrene of the uterus was the principal disease, and if it had preceded the other disorders. The woman being newly delivered gave us a clue to an explanation of the fact that the uterine disease was most probably the principal affection, and that first developed.—I will mention also, the case of gangrenous metro-vaginitis, which terminated in death, which occurred lately in our wards, and a similar case at present, of a woman in the same bed, and who besides is affected with variola. I will mention also the case of metritis, which ended fatally, and in which we found suppuration of the uterine veins.

We have had two cases of abscess of the fallopian tubes, which terminated fatally. In one case the abscess opened into the peritoneum, producing fatal peritonitis; the other was a phlegmonous tumour situated in the ovary.

Such were the most grave of our uterine diseases. We had a number of others, which I designate by the name of post-puerperal metritis, much less gave than the preceding, and which commonly terminated in resolution.

Independently of the cases of metritis connected with the puerperal state, we had six cases of simple spontaneous metritis. With regard to their etiology, we saw two cases preceded by suppression of the catamenia; two other cases followed severe fatigue; in the remaining two no appreciable cause could be discovered. All these six cases recovered. It is not rare to see metritis of this kind appear without any appreciable or known cause. There exists, as in probably all cases of essential phlegmasia, some general predisposition in the organism, which is the true cause, and which produces its influence under the slightest occasional cause.

We have had nineteen cases of uterine hemorrhage four of which followed abortion. This cause is by no means at all times easy to ascertain. Women, from different motives, which it is easy to comprehend, are often interested in hiding this circumstance, and it is sometimes not without a good deal of address, that you are able to discover it. In one case the hemorrhage seemed to have followed a suppression of the menses, caused by cold; three others seemed to be owing to some deviation of the uterus, sometimes anteriorly, and sometimes posteriorly; finally, other cases depended on cancer of the neck of the uterus, or of the body itself. We have had several cases of granular metritis, a kind of metritis very different from that which accompanies ulceration of the neck. These granular metrites may be assimilated readily to the granular alteration of the larynx, which is, as you know, very distinct from the tuberculous or syphilitic alterations of that organ. This distinction is very important; and for this reason, that the metritis of which we speak is much less dangerous than the ulcerated metritis, and is usually easily cured, by one or more cauterizations with the nitrate of silver; a treatment which, in our hands, has never failed.

### *Eruptive Diseases.*

*Variola.*—This year we have had thirty-seven cases of variola, of which five died; and three cases of very light varioloid. This word varioloid requires some explanation, in order to prevent misapprehension. It means to express an affection produced by a variolous virus, in individuals who from some peculiar circumstances, are rendered less apt to feel its effects in their worse form. By this word was wished to designate a product of the variolous virus more benign than the proper variolous virus; it is not, therefore, from any difference in the causes of these two diseases, that physicians were induced to create this new denomination, but on account of the modifications introduced into this last by the circumstances just enumerated. In fact, for a long time, it was admitted that those persons, who had once been attacked by small-pox, were not liable to it subsequently; in some way it was believed that vaccination offered the same immunity. But when, in 1816, in America, persons were attacked with secondary variola, after a first attack, and the same occured in those who had been vaccinated, and when the same phenomena were reproduced in France, it became impossible to refuse the evidence of facts, and it became necessary to admit this new eruption. It was called varioloid, whether it had appeared in persons already vaccinated, or who had suffered from a previous attack of variola, from the conviction which many physicians entertained that this secondary variola differed from the primitive one, and that another word was required to designate it. But later these facts multiplied, and it became clear to all unprejudiced minds, and whose eyes were open to evidence, that no real difference existed between the first and second affections, and that both originated from the same cause. Sometimes you see secondary variolas more severe than primitive ones. I recollect two individuals in our wards, side by side. One had a first attack of a variola; the other, one consecutive to vaccination. In the former the duration of the eruption was much shorter than the latter, and he recovered much sooner.

Hence, it is now established as a principle, that persons who have been vaccinated, are not invariably protected against an attack of small-pox, but that in general it is more benign and lighter. On this point there is at the present day general agreement. To the question, in what proportion does this occur, no satisfactory answer can be given, as our statistics on this point are not yet sufficient. In conclusion, therefore, the majority of physicians, as well as myself, consider variola and varioloid as perfectly identical.

Out of thirty-seven cases that we have had this year in the service, five died. Of these last, one was a young woman, who had œdematous laryngitis, with symptoms of suffocation, and who succumbed with this complication of diseases.

This class of affections is more common, in general, in summer than in winter. Out of the thirty-seven cases we had, twenty-four of them were in the summer semester and thirteen in the winter. This is about the ordinary proportion. The mortality, on the contrary, is greater in the winter than in the summer. There were three deaths out of thirteen cases in winter, and one only out of twenty-four cases in summer. Of these thirty-seven cases of variola, eighteen were discreet, and six were confluent and very grave, the others were intermediate. Out of the six cases of confluent small-pox, four died. With regard to sex; of these thirty-seven, twenty were men, and seventeen women, which from the greater number of men in our wards, gives about the same proportion. But the mortality was greatest among the men. In respect to age, the mortality was least between fifteen and twenty. This proportion was confirmed by the results of previous years. From them it results that the mortality was in persons between fifteen and twenty, one in eleven; and one in eight in those between twenty and thirty-five. Eleven individuals out of thirty-seven had been vaccinated and presented evident traces; seven others said that they had been, or had had a first attack of small-pox, but no trace of either assured us of the truth of the statement. Admitting, however, that of this number one-half had suffered as stated, it results that of the whole number affected with small-pox this year, that one-half had either been vaccinated, or had had small pox, which is a very considerable proportion. Out of the number indicated, eleven had not been vaccinated, or said so, and the rest, who did not recollect, showed no traces of cicatrices; nor had they had primitive variola. One only, of those who said that they had previously had variola, succumbed of confluent small-pox. Among those who had evident traces of vaccination, not one had confluent small-pox; but, on the contrary, among those where the traces were doubtful, three suffered from this form; there were also three cases of confluent small-pox, and who had not been vaccinated, and who now had small-pox for the first time. All these facts speak loudly in favour of vaccination.

In eight cases contagion was evident. The duration of the incubation was from twelve to fifteen days in four cases, which is somewhat different from the results of previous years, but which is conformable to those generally admitted. If sometimes you see cases in which the incubation seems very short, it is probably because these persons already had within them the variolous principle, before exposing themselves to any infection, (ces individus portaient, probablement, deja en eux le principe varioleux avant de s'exposer à un foyer de infection.) Suppose in fact that a person had been in contact with a variolous patient, or by some other means had been impregnated with variolous emanations, should be exposed to some subsequent contagion of the same kind, and that the day after this second exposure he is attacked with a variolous eruption, could we say that this affection was developed after a day only of incubation?

A chill is a very common prodrome of this affection. It occurred twenty times in thirty-four. Pain in the loins was quite common; we observed it eighteen times. Cephalalgia sometimes occurred; and in ten cases there was vomiting. The prodromes lasted from two to five days. Sixteen times the predromes were of very short duration, and this occurred in the confluent cases; whilst in the discreet cases the prodromes were more or less long. It is an acknowledged fact, at least in small-pox, that the disease is most severe where the prodromes are most rapid. Out of six cases of confluent small-pox, all nearly terminating in death, in five the prodromes were very short. These statistics should be borne in mind by you, as they are of great importance in prognosis.

Among those who died, one very rapidly succumbed to a complication of double pneumonia with uterine hemorrhage. Another died at the sixth day of his entrance into the hospital; he had varioloid, accompanied with repeated epistaxis, and with hemorrhagic spots on the skin, which is one of the worst forms. A third died at the end of three days, of confluent small-pox very intense, with cerebral symptoms. He had been vaccinated. Finally, another, who had not been vaccinated, died on the eleventh day; this was a very bad case.

We had six cases of varicella, all which recovered. Thus, as I remarked above, the proportion of those attacked with variola, who had been vaccinated, to those who had not been, was very great. For, including one-half of the doubtful cases, we would have nearly, out of the whole number of those attacked with small-pox, one-half vaccinated. These facts are very important, serving as they do, with a mass of facts of the same nature, collected from all parts, to throw great light upon a question of the first importance, in respect to which medical opinion is so much divided. If variola rarely occurred, and that at distant periods, it would be a much less important question. Re-vaccination would then be considered as a mere matter of precaution, and not as absolutely necessary, as many physicians think.

If a first vaccination protected you with certainty from any attack of small-pox for a period of fifteen or twenty years, a period beyond which its efficacy would be null, it would be very easy to recur, at the end of that time, to a second vaccination; this would be a very natural measure, and one dictated by common sense. But the question should be as simple as it is here stated. As it has been proposed by the Academy of Medicine, it offers many difficulties to resolve. It is my duty in this place to make known my opinion. The facts which I have given you in the course of this lecture, though few in number, *are sufficient to show the utility of re-vaccination.*

How did it happen then that when this question was agitated at the Academy of Medicine, that a number of the members disputed the opinion that I have here given, and that the majority decided against the utility of re-vaccinations? The reasons for this conduct I believe to have been the following: It was believed that if the propriety of re-vaccination was acknowledged, that it would give rise to the idea in the public mind that primary vaccinations were inefficacious, and that the acknowledged necessity of a second vaccination with the object of preventing consecutive variola, would diminish, above all in the minds of the ignorant masses, the confidence in vaccine. Certainly, if it were proved that a second vaccination was a useless and insufficient preservative against an attack of secondary small-pox, there would be some reason in abstaining from spreading among the people ideas which could disturb their confidence; but innumerable facts depose against this conclusion. It is false, too, that re-vaccination condemns primitive vaccination; it only proves that this has not an absolute and unlimited efficacy, that its preservative powers become enfeebled in time, and that they must be renewed by a new vaccination. Besides, let us suppose that this false opinion reigned among the people, ought physicians to suffer themselves to be guided by an erroneous public notion, and not by their own conviction? What inconvenience is there in a vaccinated person undergoing the operation a second time? None! it occasions even no interruption to the daily habits of the person. If after several days the vaccine pustules are not developed, you have the satisfaction of knowing that the individual is for some years, safe from any attack of variola. If, on the contrary, the vaccine succeeds, we must conclude that an attack of small-pox might sooner or later have occurred, and that by this precaution the individual will for the future be preserved from it. Let us suppose now that an individual predisposed to secondary small-pox will not be re-vaccinated, and that he is attacked with the disease, how very different is the position of this person with one who has submitted to re-vaccination. One by taking an insignificant precaution will suffer from no inconvenience, whilst the other, supposing that he has only simple discreet variola, will be obliged to remain in bed for some days, and if it should be confluent, what danger will be not run. In regard then to this there can be no doubt; the inconveniences on one side, and the advantages on the other are too evident. There is besides a reason of morality and public interest which is strongly in favour of my opinion. A person who is re-vaccinated not only preserves himself against an attack of small-pox, but the security will extend to those who might have been exposed to the contagion, had that individual instead of being re-vaccinated, had an attack of small-pox. This is an argument, I conceive, of great weight in favour of re-vaccination. Besides, by this means we may rationally anticipate sooner or later to destroy variola altogether. This consummation will indeed be consoling, and worthy in every way of our art.

There are many iudividuals who believe that they have been vaccinated, who have not been; or who think that they have had small-pox, who have not had it; for such the advantages of re-vaccination are still more evident. Thus, whatever may be the case, whether a man is certain of having been vaccinated or not, or of having had small-pox or not, it will always be advantageous for him to resort to vaccination, more especially as it occasions, as I have said before, no inconvenience. Finally, we cannot expect from vaccination more than we obtain from small-pox itself, as a preservative. Now, variola does not invariably protect one against a second attack of small-pox, even of the confluent variety; what is there then so astonishing in the fact that vaccination does not preserve indefinitely from the same accident, and that at the end of a certain time, we must recur a second time to it. I think then that after these reflexions, *we should not hesitate an instant to recur to re-vaccination, as a means of the greatest utility, and that our authorities will not be long in convincing themselves of the necessity of ordering it as a general sanitary measure.*

*Scarlatina.*

The cases of scarlet fever that we have had in the course of the present year, and which amounted to eighteen, presented sometimes very severe symptoms. Scarlet fever is commonly graver and more dangerous than measles, especially when it attacks persons of somewhat advanced age. Convalescence in this disease exacts great care, if you wish to avoid severe accidents. Convalescents from scarlet fever should not be permitted to go out before fifteen or twenty days; without this precaution there will be risk of œdema of the limbs, and other consecutive symptoms, more or less serious.

Out of the number of cases of scarlet fever mentioned above, in eight there were pains in the lumbar regions, which is remarkable, for this is a symptom peculiar to variola, (?—Ed. M. E.) It was not certain, however, that they were actual lumbar pains, for scarlatina patients suffer ordinarily at the commencement with general pains in the limbs and back.

*Measles.*

All the cases of measles terminated favourably. We remarked in all of them the characteristic sputa, white, thick, round, and swimming in a semi-opaque fluid, resembling very much the sputa of phthisis; when lecturing on the subject, I called your attention to the differential signs, which are easy to appreciate. The presence of the sputa is often very happy for the differential diagnosis between measles and scarlatina. Sometimes, you know, rubeola occurs without any eruption, which induced the ancients to admit of a variety which they called morbilli sine morbillis. It is especially in cases of this kind that you have a very good means of diagnosis, by the characteristic sputa, for wherever you meet with it, you can with certainty diagnosticate measles, even in the absence of the eruption. The attention of practitioners should be particularly called to this point.

*Erysipelas.*

We had thirty-two cases of erysipelas, of which number six died, or about one-fifth. In this number are included those cases of erysipelas that occurred as complications of other diseases. Two supervened in course of severe typhoid fevers; one case complicated a pleuro-pneumonia; a third was developed after a surgical operation; this was one of those wandering erysipelases, such as we commonly see under such circumstances. It was more frequent in women than in men, which is due probably to the greater sensibility of the cutaneous system in the former.

Spontaneous erysipelas, that which is developed under the influence of internal causes, often attacks the face, and is thence propagated to the hairy scalp; this occurred in sixteen cases. This variety of erysipelas is always grave, and frequently mortal. In all the cases, where the erysipelas was limited to the face, which were eleven in number, the disease terminated happily. Erysipelas is not so mild a disease as many physicians wish you to believe, especially erysipelas of the face, with a disposition to extend to the head. In these cases you must employ very active treatment, such as local bleedings, and even general bleeding, when the force and age of the patient permit it, and according to the intensity of the general symptoms. Whilst spontaneous erysipelas is ordinarily developed on the face, traumatic erysipelas usually appears about the seat of injury, and from thence tends to extend. You must not believe that this species of erysipelas has no other cause but an injury; there is often individual predisposition, and the wound or surgical operation acts only as a determining cause. All erysipelases can, therefore, be reduced to one variety, with regard to their future cause.

*Paris, May,* 1843.

